# Senior medical students’ awareness of radiation risks from common diagnostic imaging examinations

**Published:** 2017-12-15

**Authors:** Elena Scali, John Mayo, Savvas Nicolaou, Michael Kozoriz, Silvia Chang

**Affiliations:** 1 Department of Radiology, University of British Columbia, British Columbia, Canada

## Abstract

**Background:**

Senior medical students represent future physicians who commonly refer patients for diagnostic imaging studies that may involve ionizing radiation. The radiology curriculum at the University of British Columbia provides students with broad-based knowledge about common imaging examinations. The purpose of this study was to investigate students’ awareness of radiation exposures and risks.

**Methods:**

An anonymous multiple-choice cross-sectional questionnaire was distributed to final year medical students to assess knowledge of radiation from common diagnostic examinations and radiation-related risks following completion of the longitudinal radiology curriculum, carried out over the four years of medical training.

**Results:**

Sixty-three of 192 eligible students participated (33% response rate). The majority felt that knowledge of radiation doses of common imaging examinations is somewhat or very important; however, only 12% (N = 8) routinely discuss radiation-related risks with patients. While all respondents recognized children as most sensitive to the effects of radiation, only 24% (N = 15) correctly identified gonads as the most radiation-sensitive tissue. Almost all respondents recognized ultrasound and MRI as radiation free modalities. Respondents who correctly identified the relative dose of common imaging examinations in chest x-ray equivalents varied from 3–77% (N = 2 – 49); the remaining responses were largely underestimates. Finally, 44% (N = 28) correctly identified the excess risk of a fatal cancer from an abdominal CT in an adult, while the remainder underestimated this risk.

**Conclusion:**

Medical students acknowledge the importance of radiation-related issues to patient care. While almost all students are familiar with radiation-free modalities, many are not familiar with, and commonly underestimate, the relative doses and risks of common imaging studies. This may expose patients to increasing imaging investigations and exposure to radiation hazards.

## Introduction

It has long been recognized that ionizing radiation is a human carcinogen. Evidence for these risks is based on estimates from various sources, including atomic bomb survivors, Chernobyl nuclear accident victims, and occupational exposures such as uranium miners. In general, the effects of radiation are thought to vary according to the dose and duration of exposure and a linear, dose-dependent model is commonly accepted; as a result, there does not seem to be a threshold dose below which radiation exposure is safe.^1^–^3^

Exposure to medical radiation in the general population is increasing, which has largely been attributed to the growing and widespread use of computed tomography (CT). Indeed, since 1993 there has been a more than three-fold increase in the number of CT scans performed in the United States to approximately 70 million scans annually.^4^ The lifetime risk of developing an excess cancer as a result of an abdominal CT in an adult is estimated to be 1 in 2000.^2^ Furthermore, this figure is thought to be higher – estimated at approximately 1 in 1,000 – in radiation-sensitive populations such as children. While the risks to any individual may be small, the large numbers of patients exposed may translate into a large cumulative impact on the induction of future cancers, estimated at approximately 29,000 annually.^4^

There has been growing interest in physician knowledge of medical radiation. It seems physicians consistently underestimate doses.^5^–^14^ As a result, public interest campaigns have sought to bring greater awareness to this important issue. The fundamental underlying concern is that lack of awareness of radiation risks poses a threat to patient safety by potentially increasing their exposure to medical radiation and its attendant risks, radiation-induced cancer in particular.

### Undergraduate medical education at the University of British Columbia

The University of British Columbia (UBC) houses the largest medical school in Canada. The overriding objective of the medical curriculum is broad-based training for competency in general practice. Over the last several years, the undergraduate radiology curriculum has been formalized and integrated into the medical education curriculum, which places an emphasis on radiologic anatomy, introduction of imaging modalities and basic image interpretation. This mandatory curriculum is delivered by staff radiologists, radiology fellows, and residents longitudinally throughout undergraduate medical training. In addition, a series of summary lectures are provided to final year medical students during the last few months of their training. Over the four years of medical school, this amounts to approximately 40 hours of direct, large group teaching, including 25 hours of radiology anatomy and 15 hours of clinical radiology. This curriculum is the only teaching of radiology that all medical students receive.

Importantly, the radiology curriculum includes specific radiation safety objectives (Table 1). This reflects the recognition of radiology as a crucial part of clinical medicine with a strong contribution towards patient care. Similarly, in recent years radiology-specific objectives have also been integrated into nation-wide medical licensing examinations. Given that this curriculum has been recently introduced, this study was undertaken as a baseline assessment of students’ knowledge following completion of the longitudinal radiology curriculum, from which adjustments to the curriculum content can be made.

## Purpose

The purposes of this study were to assess 1) senior medical students’ knowledge of radiation exposures from common diagnostic imaging studies, 2) senior medical students’ knowledge of radiation risks, including radiation-sensitive tissues and populations, and 3) the need for additional educational resources to improve undergraduate medical education on radiation-related issues.

## Methods

The study population consisted of final (4^th^) year medical students (n = 192) at the Vancouver-Fraser campus of the University of British Columbia in April 2015. An anonymous multiple-choice questionnaire was administered to them during a classroom-based radiology lecture towards the end of their medical school training, following completion of the longitudinally integrated radiology curriculum. Responses were obtained in real time using electronic hand held audience response devices and students were given 60 seconds to submit each response. The questions were based on material taught throughout the curriculum. A twenty-one item questionnaire was administered to the students. The first section of the questionnaire (seven questions) addressed the students’ demographic background, including perceived knowledge of radiology and radiation-related issues. The second section of the questionnaire (thirteen questions) consisted of closed-ended, multiple choice questions addressing specific knowledge of the absolute and relative radiation doses (in terms of chest x-ray equivalents) from commonly ordered diagnostic studies, which are common methods of assessing radiation doses used in the literature, a concept introduced in the curriculum. The last question asked about delivering content on imaging risks (lecture, tutorial or workshops, and web-based material).

For the correct answer we used the average effective dose estimates as well as the effective dose from background radiation provided in the literature.^15^ Mean scores were calculated for each respondent for each of the 11 questions for which there was a single right answer (questions 8 through 17 and question 19 in Appendix A). To determine the statistical significance of the difference between subgroups, these means were compared using a one-way analysis of variance. To avoid small sample sizes in the subgroups based on students’ residency of choice, medical and surgical specialties were grouped together. Statistical significance was defined at a p-value (two-sided) of less than 0.05. University of British Columbia Research Ethics Board approval was obtained for this study and implied consent was assumed if the survey was completed.

## Results

Sixty-three of 192 eligible students who attended the lecture responded, for a response rate of 33%. The mean age of respondents was 27 years old (range: 20 – 34 years). There were 43% (N = 27) male and 57% (N = 36) female respondents, which reflects the composition of the class overall. Of the residency programs to which respondents had applied, 35% (N = 22) applied to family medicine, 35% to medical specialties (N = 22), 19% to surgical specialties (N = 12) and remaining 11% to all other specialties (N = 7).

The majority of respondents (62%, N = 39) reported feeling not very confident or not at all confident, while 38% (N = 24) reported feeling very or somewhat confident. Approximately half of respondents felt that their knowledge of radiology was similar to other areas (46%, N = 29), while the remainder reported their knowledge was worse or inferior.

Most students (92%, N = 56) responded that knowledge of the radiation dose of common imaging examinations is somewhat or very important. In spite of this, however, only 12% (N = 8) of respondents always or often discuss radiation-related risks when referring patients for imaging examinations; 76% sometimes (N = 48) and 12% (N = 8) never address these risks.

Eighty-six percent (N = 54) of respondents correctly recognized that CT is most responsible for medical radiation received by the population. While all respondents recognized children as the population most sensitive to the effects of radiation, only 24% (N = 15) correctly identified gonads as the most radiation-sensitive tissue. Forty-four percent (N = 28) of the respondents correctly identified the risk of inducing a fatal cancer from an abdominal CT in an adult while the remainder underestimated this risk.

Forty-four percent (N = 28) correctly identified (or guessed) the absolute dose of a chest x-ray (in milliSieverts) and 33% (N = 21) correctly identified the relative dose of a chest x-ray compared to background radiation. The remainder of respondents overestimated the dose of a chest x-ray in both absolute and relative terms. All respondents identified ultrasound and 97% (N = 61) and MRI as radiation-free modalities. The proportion of students able to correctly identify the relative dose of common imaging studies in chest x-ray equivalents varied from 3–77% (N = 2–49); the remainder were largely underestimates (Table 2).

The mean overall score was 5.96 out of a possible 11 and scores ranged from 4 to 9. The subgroup analysis compared mean scores according to age, sex and residency program of choice as well as self-reported knowledge of radiology, confidence of knowledge of radiation doses of common imaging studies and importance of knowledge of radiation doses. No statistically significant differences were found among any of these subgroups.

The majority of respondents (92%, N = 56) agreed that workshops, tutorials or web-based learning modules could be used to increase awareness of radiation risks.

## Discussion

As future physicians, medical students must fully understand the magnitude and implications of radiation doses and risks that accompany common imaging studies for which they will refer their patients. Indeed, the importance of this issue is reflected in the drive towards appropriate use of medical imaging as well as the widespread adoption of radiation safety principles including as low as reasonably achievable (ALARA). There is growing concern that this general lack of awareness makes it difficult, if not impossible, to properly inform patients of the risks and benefits of a given study. Moreover, the widely observed underestimation of radiation dose may lead to more imaging studies than are necessary, which is not without harm to the patient.

Studies of physicians’ knowledge of radiation exposure and associated risks have demonstrated consistent underestimation of doses associated with various imaging studies.^5^–^7^,^9^,^10^,^12^,^16^–^19^ For example, a study of 130 doctors in the United Kingdom found that 97% underestimated the radiation dose (in chest x-ray equivalents) of common imaging studies.^12^ The literature also demonstrates a consistent inability to correctly identify the excess cancer risk as a result of an abdominal CT in an adult: indeed, only 12.5% of 240 doctors surveyed in the UK were correct.^16^ Similarly, a survey of 331 Australian medical students revealed that 59% underestimated this risk.^10^ Discussion around the risks and benefits of imaging studies that involve ionizing radiation does not take place consistently.^6^,^13^,^17^,^18^ For example, 7% of the 76 patients who underwent abdominal CT scan in the emergency department of an American medical centre reported being informed of the risks and benefits of the study.^18^

Despite public awareness campaigns such as Image Gently, similar results have been found for radiation-sensitive populations such as children.^20^,^21^ Indeed, a study of 220 Canadian pediatricians demonstrated underestimation of radiation dose by 87% of respondents; furthermore, only 6% were able to correctly identify the lifetime excess cancer risk.^13^ Similarly, an American study of 147 pediatric surgeons revealed that 77% did not discuss specific risks of CT scan with their patients and even more respondents, 83%, did not address potential cancer risks.^17^

The literature also demonstrates a substantial minority of respondents unable to identify modalities that involve the use of ionizing radiation.^5^–^7^,^9^,^10^,^12^,^16^,^19^ For example, 5% and 15% of the 80 intern and physician respondents in a Hong Kong study thought that abdominal ultrasound and abdominal MRI, respectively, involve the use of ionizing radiation.^6^ Similar trends are seen amongst medical students: an Australian study of 331 medical students revealed that 11% and 26% incorrectly believed that ultrasound and MRI, respectively, involve the use of ionizing radiation.^10^

In general, variability in the knowledge of radiation risks and doses has been found based on both area of specialty and years of experience. For example, radiologists tend to be more knowledgeable than non-radiologists and more experienced physicians tend to perform better than their junior colleagues.^5^,^6^,^9^,^10^,^16^,^18^ Radiation awareness among medical students demonstrates a similar trend: an Irish study of 670 medical students who received clinical radiology training over their five-year program found incremental improvement of students’ knowledge throughout increasing years in medical school and/or exposure to radiology teaching.^5^ Taken together, these findings underscore the importance of instruction on radiation protection, which has been shown to produce a statistically significant improvement on knowledge of radiation risks.^5^,^19^,^22^

Limitations of this study include the small sample size, final year medical students who chose to voluntarily participate in this single centre study. In addition, the radiology component of the undergraduate medical curriculum has been recently introduced and, as a result, ongoing refinements are being made to the content of the curriculum in line with the stated objectives. It is inherently difficult to generalize the effective doses of imaging studies; as a result, the “correct” values presented above represent averages taken from the literature and effective doses in clinical practice may vary by an order of magnitude as a result of various factors including dose reduction techniques. Finally, the scope of the study was limited to core principles of radiation safety; as such, many important areas, such as imaging the pregnant patient have not been addressed.

Deficiencies in medical students’ knowledge of radiation-related issues have been previously described in the literature. Given that the University of British Columbia houses the largest medical school in the country and has recently formalized and integrated radiation protection principles into the undergraduate medical curriculum, we feel that this work is both important and timely. In order to improve upon some of the deficiencies identified by this study, we plan to develop additional resources for senior medical students with a view to improve their knowledge and, thereby, patient care. Moreover, the widespread lack of knowledge among medical students begs the question as to whether radiation protection principles should be a more prominent component of medical education for all physicians, beginning even in medical school, both in British Columbia and across the country.

## Figures and Tables

**Table 1:** Radiation-related objectives of the UBC undergraduate medical curriculum

1.	Understand the differences between imaging modalities including advantages, limitations, and radiation exposure from plain film radiography, fluoroscopy, CT, ultrasound, MRI, and nuclear medicine.
2.	Understand the risks associated with radiation exposure, specifically haematological and solid organ malignancies, local skin effects, and teratogenic effects.
3.	Appreciate the chest x-ray equivalents of common examinations for various imaging modalities.
4.	Recognize methods to reduce radiation exposure such as reduction in unnecessary examination, reducing dose on CT protocols, reducing exposure time, and use of non-ionizing radiation modalities such as ultrasound and MRI.

**Table 2:** Percentage of respondents who correctly identified, underestimated or overestimated the relative doses in chest x-ray equivalents from 6 commonly requested studies.

	% underestimate	% correct	% overestimate
Abdominal x-ray	91	3	5
CT abdomen and pelvis	24	77	0
Abdominal ultrasound	0	100	0
Spine MRI	0	96	2
V/Q scan	44	46	9
Bone scan	90	10	0

## Appendix A. Radiation Risks from Common Diagnostic Imaging Examinations: Questionnaire

Please select one answer by choosing the appropriate checkbox.

1. What is your sex:
  ▪ Male
  ▪ Female
2. What age group do you belong to?
  ▪ <20
  ▪ 20–24
  ▪ 25–29
  ▪ 30–34
  ▪ 35–39
  ▪ >40
3. What residency training program have you matched to?
  ▪ Family Medicine
  ▪ Surgical specialties
  ▪ Medical specialties
  ▪ All other specialties
4. How does your knowledge of radiology compare with other subjects?
  ▪ Superior
  ▪ Better
  ▪ Similar
  ▪ Worse
  ▪ Inferior
5. How confident are you in your knowledge of the ionizing radiation dose of common diagnostic imaging techniques?
  ▪ Very confident
  ▪ Somewhat confident
  ▪ Not really confident
  ▪ Not at all confident
6. How important would you rate having knowledge of the radiation dose of common radiological investigations?
  ▪ Very important
  ▪ Somewhat important
  ▪ Not really important
  ▪ Not at all confident
7. How often do you discuss radiation-related issues, including long-tern risks, with patients when offering radiological investigations?
  ▪ Always
  ▪ Often
  ▪ Sometimes
  ▪ Never
8. What is the approximate radiation dose, in milliSieverts, of a chest x-ray?
  ▪ 0.02
  ▪ 0.2
  ▪ 2
  ▪ 20
  ▪ Don’t know
9. In a chest x-ray, the radiation dose is the same as natural background radiation received in less than
  ▪ 1 week
  ▪ 1 month
  ▪ 6 months
  ▪ 1 year
  ▪ Greater than one year
  ▪ Don’t know
10. What is the dose, in chest x-ray equivalents, for an abdominal x-ray?
  ▪ 0
  ▪ 1–5 times
  ▪ 5–10 times
  ▪ 10–50 times
  ▪ 50–300 times
  ▪ Over 300 times
11. What is the dose, in chest x-ray equivalents, for a CT of the abdomen and pelvis?
  ▪ 0
  ▪ 1–5 times
  ▪ 5–10 times
  ▪ 10–50 times
  ▪ 50–300 times
  ▪ Over 300 times
12. What is the dose, in chest x-ray equivalents, for an abdominal ultrasound?
  ▪ 0
  ▪ 1–5 times
  ▪ 5–10 times
  ▪ 10–50 times
  ▪ 50–300 times
  ▪ Over 300 times
13. What is the dose, in chest x-ray equivalents, for a MRI of the lumbar spine?
  ▪ 0
  ▪ 1–5 times
  ▪ 5–10 times
  ▪ 10–50 times
  ▪ 50–300 times
  ▪ Over 300 times
14. What is the dose, in chest x-ray equivalents, for a V/Q scan?
  ▪ 0
  ▪ 1–5 times
  ▪ 5–10 times
  ▪ 10–50 times
  ▪ 50–300 times
  ▪ Over 300 times
15. What is the dose, in chest x-ray equivalents, for a bone scan?
  ▪ 0
  ▪ 1–5 times
  ▪ 5–10 times
  ▪ 10–50 times
  ▪ 50–300 times
  ▪ Over 300 times
16. Which one of the following groups is the **most** sensitive to the effects of radiation?
  ▪ Children
  ▪ Adolescents
  ▪ Adults
  ▪ Elderly
  ▪ Don’t know
17. Of the organs listed, which is the **most** sensitive to radiation?
  ▪ Thyroid
  ▪ Breast tissue
  ▪ Gonads
  ▪ Kidney
  ▪ Don’t know
18. Which of the following increases the lifetime risk of developing cancer? Select all that apply.
  ▪ Ultrasound
  ▪ Plain films
  ▪ CT
  ▪ MRI
19. Medical imaging accounts for approximately 15% of the radiation dose received by the population. Which of the following is the **most** responsible for this radiation dose?
  ▪ Ultrasound
  ▪ Plain films
  ▪ CT
  ▪ MRI
  ▪ Don’t know
20. What is the risk of inducing fatal cancer from an abdominal CT scan in an adult?
  ▪ 1/200
  ▪ 1/2,000
  ▪ 1/20,000
  ▪ 1/200,000
  ▪ Don’t know
21. Which of the following educational methods do you think would help to raise awareness of radiation-related issues?
  ▪ Lectures
  ▪ Tutorials or workshops
  ▪ Web-based learning modules
